# Stability of buried carbon in deep-ploughed forest and cropland soils - implications for carbon stocks

**DOI:** 10.1038/s41598-017-05501-y

**Published:** 2017-07-14

**Authors:** Viridiana Alcántara, Axel Don, Lars Vesterdal, Reinhard Well, Rolf Nieder

**Affiliations:** 1 Thünen Institute of Climate-Smart Agriculture, Bundesallee 50, Braunschweig, 38116 Germany; 2 Food and Agriculture Organization of the United Nations, Viale delle Terme di Caracalla, 00153 Rome, Italy; 3 Department of Geosciences and Natural Resource Management, University of Copenhagen, Rolighedsvej 23, DK-1958 Frederiksberg C, Denmark; 4 Institute of Geoecology, Technische Universität Braunschweig, Langer Kamp 19c, Braunschweig, 38106 Germany

## Abstract

Accumulation of soil organic carbon (SOC) may play a key role in climate change mitigation and adaptation. In particular, subsoil provides a great potential for additional SOC storage due to the assumed higher stability of subsoil SOC. The fastest way in which SOC reaches the subsoil is via burial, e.g. via erosion or deep ploughing. We assessed the effect of active SOC burial through deep ploughing on long-term SOC stocks and stability in forest and cropland subsoil. After 25–48 years, deep-ploughed subsoil contained significantly more SOC than reference subsoils, in both forest soil (+48%) and cropland (+67%). However, total SOC stocks down to 100 cm in deep-ploughed soil were greater than in reference soil only in cropland, and not in forests. This was explained by slower SOC accumulation in topsoil of deep-ploughed forest soils. Buried SOC was on average 32% more stable than reference SOC, as revealed by long-term incubation. Moreover, buried subsoil SOC had higher apparent radiocarbon ages indicating that it is largely isolated from exchange with atmospheric CO_2_. We concluded that deep ploughing increased subsoil SOC storage and that the higher subsoil SOC stability is not only a result of selective preservation of more stable SOC fractions.

## Introduction

Soil organic carbon (SOC) is currently receiving increasing attention in science and politics due to its great potential to act as a sink for atmospheric CO_2_ and thus mitigate climate change^1^. SOC may also help to adapt to climate change because of its beneficial effect on soil structure, water-holding capacity and nutrient retention^2^. SOC sequestration is generally achieved, when C inputs outbalance C losses through decomposition. Apart from land use conversions such as converting cropland to forest, SOC accrual can be achieved through implementation of certain management practices including conservation agriculture, cover crop cultivation and mulch farming, among many others^3, 4^. Currently, most SOC sequestration management measures are based on assessment of the increase in SOC content in the top layers of the soil. However, over half of world’s total SOC is located below 30 cm depth, in the subsoil^5, 6^. Although SOC concentration decreases with depth, SOC stocks in subsoil are mostly greater than in topsoil because subsoil has a larger soil mass, and thus larger potential storage capacity, than topsoil.

Subsoil OC is reported to be more stable than SOC near the soil surface, a trend inferred from increasing apparent radiocarbon age with depth, indicating that deep SOC has prevailed in soils and has been excluded from exchange with the atmosphere for centuries to millennia^7, 8^. It has been widely suggested that subsoil has great potential to store additional SOC than topsoil^9, 10^ because of the large number of unsaturated mineral surfaces^11^ and environmental conditions that slow SOC mineralisation^12^ (e.g. more constant moisture and temperature regime or oxygen limitation). Additional carbon inputs have been observed to decompose more slowly in subsoil than in topsoil^13^. These slower SOC mineralisation rates in subsoil have been attributed to the lower SOC content, which results in a lower density of decomposing microorganisms and thus a lower possibility of any SOC present being mineralised^14^. Also lower oxygen concentration in subsoils and less disturbance via drying-rewetting and freezing-thawing cycle and via tillage has been discussed as reasons for higher SOC stability in subsoils^12^.

Carbon enters subsoil mainly with aboveground and belowground litter, dead roots and root exudates, dissolved and particulate organic carbon (OC) transported via large pores or through biological soil reworking (bioturbation)^10^. However, deeper burial of C-rich soil material, e.g. by deposition following erosion, generally leads to a long-term increase in landscape-scale SOC stocks^15^. Active anthropogenic SOC burial has rarely been studied, but is occasionally carried out through deep ploughing of agricultural and forest land. Although deep ploughing is not one of the most common agricultural practices, its implications on SOC sequestration have been analysed in the present study because of the direct impact on subsoil it entails.

Deep ploughing is a land management operation performed mostly only once, with the purpose of loosening the subsoil, enhancing water infiltration and root penetration capacity and thus improving plant growing conditions. Further subsoil management techniques, such as deep mixing, deep ripping, deep rototilling and deep loosening are performed more than once or even on a regular basis. In this study, we studied solely sites that has been deep ploughed only one time. Through the action of deep ploughing, SOC-rich topsoil is buried at 60–120 cm depth and SOC-poor subsoil material is brought up to the surface. The latter also transports nutrients from the less weathered subsoil to the surface, making them more easily available to plants. As a preparation measure for afforestation (active forest establishment on previous non-tree land that has not supported trees for several decades), deep ploughing leads to a higher survival rate of planted trees because of better weed control, with weed seeds being buried, and better water availability when the OC-rich A horizon with high water-holding capacity is placed deeper in the soil^16^.

Deep ploughing was promoted in Europe after the invention of the steam plough in the late 19th century^17, 18^, enabling ploughing depths of 60 to 120 cm since 1950. Peatlands were largely frequently deep ploughed to facilitate their cultivation, but in this study we focus only on mineral soils. Deep ploughing was performed in heathland soils to break up the hard pan of Podzols in order to facilitate making them cultivatable. The pursued effects of deep ploughing also related to erosion reduction in Luvisols through mixture of clay-rich subsoil material to the silt-dominated topsoil^19^. Deep ploughing has become less common since the 1970s. Nevertheless it is still applied for hard pan or plough pan break-up, in order to enlarge the rooting zone or as a preparation measure for afforestation in several countries: Canada^20^, Denmark^16^ The Netherlands^21^, Sweden^22^ China^23^ and USA^24^. In Northern Germany around 10% of the croplands have been deep ploughed during the last 60 years.

Deep ploughing of cropland is reported to be a very effective long-term SOC sequestration measure^25^. At 10 cropland sites on mineral soils, deep ploughing led to a 42% increase in SOC stocks after 45 years because carbon in the buried topsoil was not entirely mineralised, if at all, and additional SOC was continuously accumulated in the “newly formed” topsoil mixed with subsoil material.

Because deep ploughing translocates large amounts of SOC to the subsoil and also facilitates deep rooting, subsoil SOC stocks can be expected to increase over the long-term, including in forests. Deep ploughing of forest soil also leads to burial of the organic layer that forms on top of the mineral soil – the forest floor - with its additional carbon. Rooting patterns are also different in forest compared with cropland with deeper and more roots in forest soil^26^. In the present study, we investigated the effect of carbon burial through deep ploughing in forest and cropland soils that were deep ploughed 25 to 53 years before sampling. In addition, because biomass removal is four-fold more intense for crops than for forests^27^, SOC in the newly formed topsoil of deep-ploughed soil can be expected to accumulate faster in forest soil than in cropland. On the other hand, fertilisation, liming and tillage of cropland may stimulate SOC accumulation in topsoil of the deep-ploughed arable soils. The following hypotheses were tested in this study:

1. SOC stocks increase on a long-term basis after deep ploughing compared with non-deep-ploughed reference soil. This SOC accrual is greater in forests than in cropland.
2. Buried SOC is more stable to mineralisation than non-buried SOC in reference topsoils.

## Results

### Depth distribution of SOC contents and stocks

**Figure 1:**
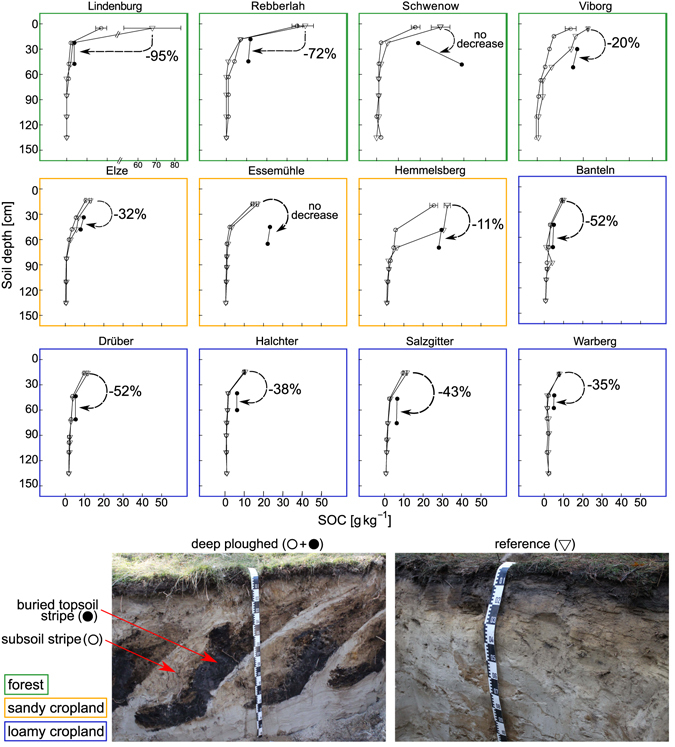
Depth distribution of mean SOC content in deep-ploughed (⚬ •) and reference plots ($\nabla$). Topsoil SOC content in soil profiles and cores (N = 6). Deep-ploughed plots consist of alternating buried topsoil (•) and subsoil stripes (⚬). Dashed arrows and percentages indicate the relative difference between average topsoil SOC and average SOC in buried topsoil stripes.

Buried topsoil stripes in deep-ploughed forest soil had higher SOC contents (16 ± 4 g C kg^−1^) than the corresponding depth layer in adjacent subsoil stripes (4 ± 1 g C kg^−1^) and the reference subsoil (7 ± 2 g C kg^−1^) (Fig. 1). This was also observed for the cropland soils studied (16 ± 5 g C kg^−1^ in buried topsoil stripes, 3 ± 0.4 g C kg^−1^ in the adjacent subsoil stripes and 4 ± 2 in the reference subsoil). At two sites, SOC content in the buried topsoil was comparable to that in the reference topsoil: for the Schwenow forest site these values were 29 ± 10 g C kg^−1^ in the buried topsoil and 26 ± 4 g C kg^−1^ in the reference topsoil, and for the Essemühle cropland site they were 17 ± 6 g C kg^−1^ in the buried topsoil and 23 ± 1 g C kg^−1^ in the reference topsoil. At all other sites, SOC content in the buried topsoil stripes was reduced by 11% (Hemmelsberg cropland site) to 95% (Lindenburg forest site) in comparison with the non-buried reference topsoil. Assuming that, before deep ploughing, the SOC content in the buried topsoil stripes was similar to that in the current reference topsoil, the fastest decrease in SOC content in buried topsoil stripes following deep ploughing was at the Lindenburg (mean annual decrease 2.6% over 37 years) and Rebberlah (mean annual decrease 2% over 36 years) forest sites.

Newly formed topsoil on deep-ploughed cropland soil (57 ± 5 Mg C ha^−1^) had 8% lower SOC stocks than the reference topsoil (64 ± 6 Mg ha^−1^, p = 0.02). An even larger difference of 37% was observed in forest soils (20 ± 1 in the topsoil of the deep-ploughed plots and 32 ± 3 Mg ha^−1^ in the topsoil of the reference plots, p < 0.0001). Forest floor followed the same trend, but differences were only significant in the F + H-horizons (6 ± 1 Mg ha^−1^ in deep-ploughed and 15 ± 3 Mg ha^−1^ in reference plots, p = 0.03). On average, the difference between topsoil SOC stocks in deep-ploughed and reference soils relative to the number of years since deep ploughing was −0.16 Mg ha^−1^ yr^−1^ in cropland and −0.31 Mg ha^−1^ yr^−1^ in forest soil. Moreover, the nitrogen (N) stocks in topsoil of deep-ploughed soil were substantially lower than in reference soil in forests, while at five out of eight cropland sites studied, the topsoil of deep-ploughed soils contained more N than reference topsoil (Supplementary Table 1).

**Figure 2:**
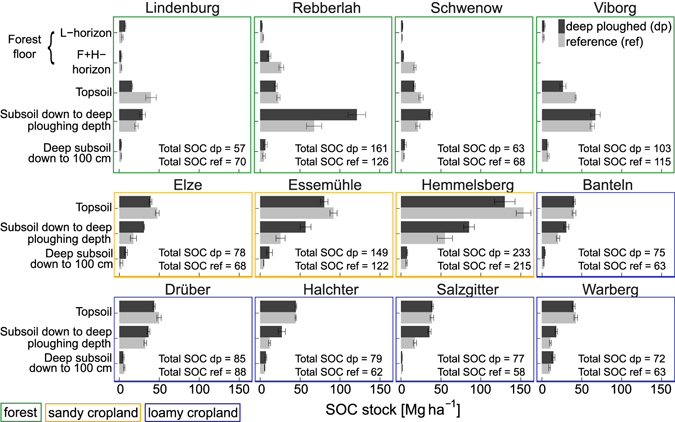
SOC stocks at different soil depth increments in deep-ploughed and reference plots. Bars represent average SOC stocks in soil cores (n = 5), whiskers show standard error. Subsoil and buried topsoil stripes were not sampled separately. Total SOC stock sums for forest sites include forest floor.

Total SOC stocks down to 100 cm were significantly greater (p < 0.0001) in deep-ploughed (105 ± 8 Mg C ha^−1^) than in reference cropland soil (92 ± 8 Mg C ha^−1^, Fig. 2). Below 30 cm down to deep-ploughing depth, deep-ploughed cropland subsoil (40 ± 3 Mg C ha^−1^) contained 67 ± 17% more SOC than reference subsoil (24 ± 3 Mg ha^−1^, p < 0.0001). In contrast, total SOC stocks in deep-ploughed forest soil (including forest floor) were not significantly greater than in reference soil (103 ± 11 and 105 ± 9 Mg ha^−1^, respectively, p = 0.2). However, SOC stocks in forest subsoil were 49 ± 25% greater in deep-ploughed than in reference soil (64 ± 9 and 43 ± 6 Mg ha^−1^, respectively, p = 0.0002). The SOC stocks below the deep-ploughed horizon did not differ between treatments within forest or cropland sites.

### Potential SOC mineralisation

**Figure 3:**
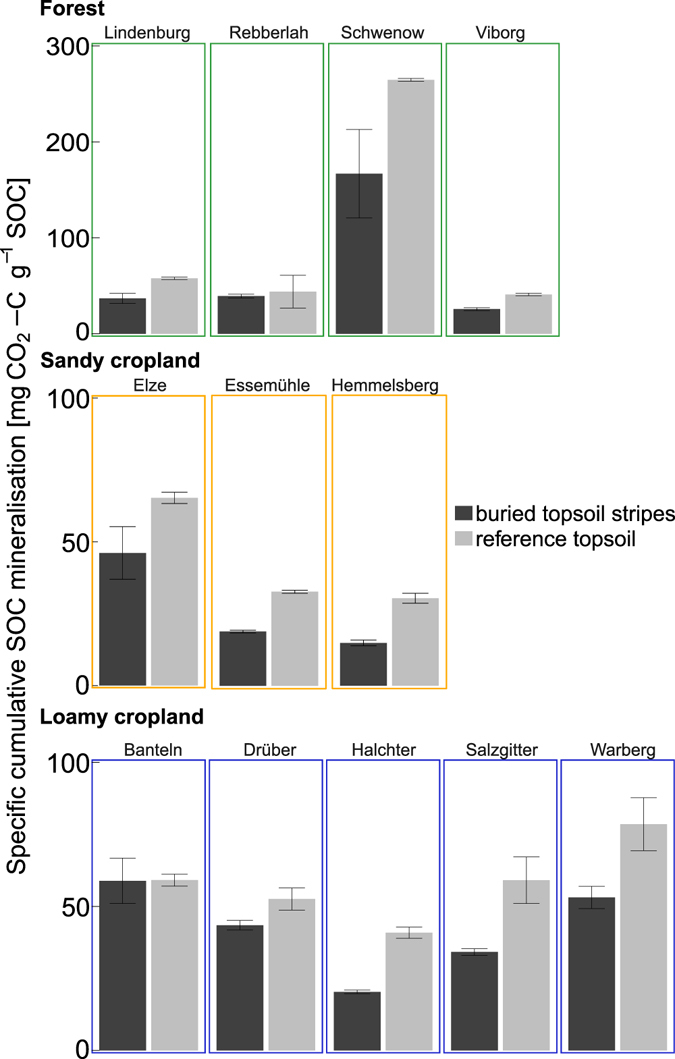
Specific cumulative SOC mineralisation after one year of incubation. Bars represent mean values from laboratory replicates (n = 3), whiskers show standard error.

Buried SOC stability was assessed through one-year incubation experiments, which enabled comparison of SOC turnover in buried topsoil stripes and reference topsoil under standardised laboratory conditions eliminating possible oxygen or water limitations. The fraction of mineralised SOC was 32% lower in incubated buried topsoil than in reference topsoils (Fig. 3, p < 0.0001). Forest soils had the highest mineralisable SOC fraction, both in buried topsoil stripes (56 ± 13 mg CO_2_-C g^−1^ SOC) and in reference topsoil (77 ± 22 mg CO_2_-C g^−1^ SOC). Sandy cropland buried topsoil stripes (27 ± 4 mg CO_2_-C g^−1^ SOC) and reference topsoil (40 ± 6 mg CO_2_-C g^−1^ SOC) had the lowest mineralisable SOC fraction. There was a weak positive correlation between the relative difference in specific cumulative SOC mineralisation and the relative difference in SOC content for the buried topsoil stripes and the reference topsoil (Rho = −0.6, p = 0.04).

### Carbon input and SOC fractions

**Figure 4:**
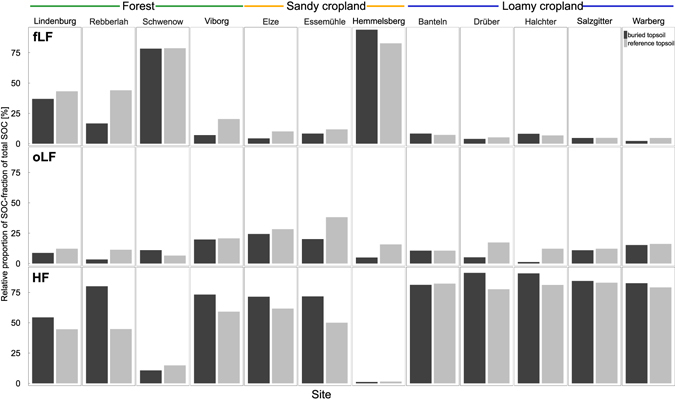
Relative proportion of free light fraction (fLF), occluded light fraction (oLF) and heavy fraction (HF) of total SOC in buried topsoil stripes and reference topsoil.

The relative distribution of different SOC fractions provided information about the degree of stabilisation of carbon in the buried topsoil stripes and the reference topsoil. The free light fraction (fLF) is usually the youngest and most labile SOC fraction. However, the fLF fraction was not consistently lower in buried topsoil stripes than in reference topsoil, but rather ranged between 65% lower and 19% higher indicating that topsoil burial with deep ploughing did not always resulted in losses of fLF (Fig. 4). Similarly, we found buried SOC stabilisation to be not a result of SOC aging and selective preservation since the correlation between the burial effect on specific SOC mineralisation rates and the burial effect on fLF mass was very weak and not significant (Rho = −0.3, p = 0.3). Also the occluded light fraction (oLF) was between 90% lower and 70% higher in buried topsoil stripes than in the reference topsoil. The most stable heavy fraction (HF) did not consistently contain most of the SOC in buried topsoil. Instead, SOC in the HF was between 28% lower and 79% higher in the different sites in buried topsoil compared with reference topsoil.

**Figure 5:**
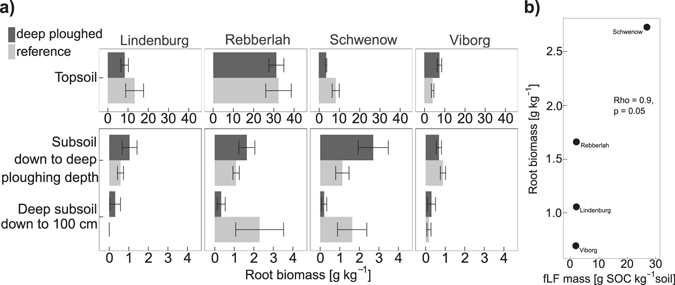
(**a**) Root biomass at different soil depth increments in deep-ploughed and reference forest soil. Bars represent average root biomass in soil cores (n = 5), whiskers show standard error. (**b**) Correlation between root biomass in deep-ploughed subsoil and fLF mass calculated as Spearman’s rank correlation.

Fine root biomass was used as an indicator for carbon input as labile SOC. In general, root biomass was 10 to 100 times lower in cropland than in forest soil (Fig. 5a). Throughout the entire soil profile, root biomass in cropland did not differ between deep-ploughed and reference plots. In contrast, root biomass in forest topsoils tended to be lower in deep-ploughed plots than in reference topsoil (13 ± 3 and 15 ± 3 g kg^−1^, respectively). Deep-ploughed forest subsoil had 65% higher root biomass than reference subsoils (1.5 ± 0.3 and 0.9 ± 0.11 g kg^−1^, respectively, p = 0.04). At the Viborg forest site only, relative root mass was very similar in subsoil of both plots. Root biomass in the deep-ploughed subsoil was highly correlated to the fLF content (Fig. 5b).

### Radiocarbon content

**Figure 6:**
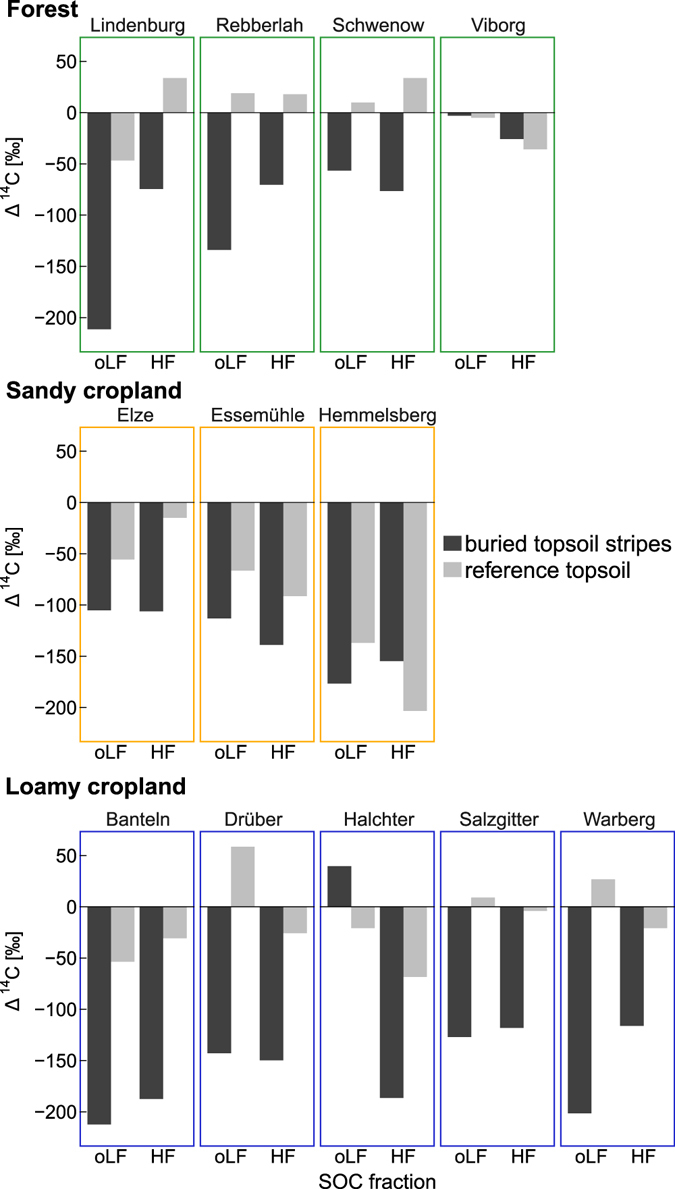
Δ^14^C values obtained for occluded light fraction (oLF) and heavy fraction (HF) of SOC in buried topsoil stripes and reference topsoil. Positive values signify that nuclear test- derived ^14^C was incorporated into the SOC fraction (young apparent ^14^C age). Negative values indicate that soil fraction carbon exchange with the atmosphere has been slow and that significant radioactive decay has occurred (old apparent ^14^C age)^35^.

The radiocarbon (^14^C) content of the SOC fractions oLF and HF provided information about the mean residence time of carbon in the soil. Longer residence times are generally characterised by a low ^14^C content. The oLF had higher ^14^C content than HF indicating faster turnover of the oLF (−71‰ in the oLF and −76‰ in the HF). The oLF of the buried topsoil stripes had was depleted in Δ^14^C compared to reference topsoils at all sites except the Viborg forest site and the Halchter loamy cropland site (Fig. 6, −120 ± 23‰ in the Viborg forest site and −22 ± 15‰ in the Halchter loamy cropland site). The ^14^C content in the oLF was lower in buried topsoil stripes than in reference topsoil, by 96‰ in forest, 45‰ in sandy cropland and 133‰ in loamy cropland. This pattern of lower ^14^C content in buried SOC compared with reference topsoil was also observed for HF (−117 ± 14‰ in the buried SOC and −34 ± 18‰ in the reference topsoil SOC). The ^14^C content in HF was lower in buried topsoil stripes than in reference topsoil with on average 74‰ in forests, 30‰ in sandy croplands and 121‰ in loamy croplands. For both fractions, in buried topsoil stripes as well as in reference topsoil, Δ^14^C was higher in forests than in croplands (Fig. 6). Forested reference topsoil was mostly enriched in ^14^C compared to preindustrial reference, indicating the influence of ^14^C from nuclear weapons testing since the 1960s (oLF: −6 ± 15, HF: 12 ± 16‰).

## Discussion

The first part of this section will look into the effects of deep ploughing on SOC stocks in subsoils, topsoils and the full assessed first meter of the soil. The second part will look discuss the stability of buried SOC and its possible mechanisms.

The SOC stocks in subsoil were significantly greater in deep-ploughed soil than in reference plots (Fig. 2). The data revealed that topsoil SOC was partly preserved upon burial in subsoil up to 53 years ago in both, forest and cropland soils (Table 1). In colluvial deposition areas under cropland, topsoil buried to 30–70 cm depth has been found to contain more SOC 50 years after burial than the corresponding topsoil in upslope areas^28^. In subsoil environments, SOC mineralisation presumably takes place over a timescale of centuries, with 50% of the buried SOC mineralised after ca. 250–300 years^29^. On a short-term basis (5 years), farmyard manure buried to 60 cm depth has been observed to at least double the SOC content in subsoil by preserving approximately 80% of the buried SOC^30^. This has been attributed to the lack of physical disturbance in deeper soil layers, e.g. by freeze-thawing, drying-rewetting cycles or regular tillage.

**Table 1:** Site characteristics. Soil properties refer to the topsoil in reference plots (texture: n = 1, pH: n = 6). Cropland data as published previously^25^.

Land use	Site	Deep ploughing year	Years between deep ploughing and sampling	Deep ploughing depth [cm]	Dominant tree species or crops	Soil type	Sand [%]	Silt [%]	Clay [%]	pH	Former land use
Forest	Lindenburg	1977	37	60	*Pinus sylvestris, Quercus robur, Fagus sylvatica*	Spodic Cambisol	86	9	5	4.5 ± 0.3	Forest
Forest	Rebberlah	1978	36	58	*Pinus sylvestris, Picea abies*	Lamellic Podzol	84	11	5	3.1 ± 0.1	Heathland/Forest
Forest	Schwenow	1961	53	60	*Quercus rubra* (deep ploughed), *Pinus sylvestris* (reference)	Haplic Podzol	89	11	0	4.4 ± 0.7	Heathland
Forest	Viborg	1989	25	62	*Quercus robur, Pinus sylvestris* (during first 24 years)	Haplic Cambisol	86	9	5	4.8 ± 0.2	Heathland, Cropland
Cropland	Elze	1968	46	55	Oilseed rape, rye, potato	Dystric Cambisol	84	12	4	5.3 ± 0.1	Cropland
Cropland	Essemühle	1968	46	75	Oilseed rape, potato, barley, rye, maize	Dystric Cambisol	88	8	4	4.8 ± 0.1	Heathland
Cropland	Hemmelsberg	1978	36	80	Oilseed rape, rye, potato	Dystric Cambisol	94	3	3	5.3 ± 0.05	Peatland
Cropland	Banteln	1965	48	85	Sugar beet, wheat, maize	Haplic Luvisol	5	82	13	6.6 ± 0.1	Cropland
Cropland	Drüber	1966	48	87	Oilseed rape, wheat, barley	Haplic Luvisol	3	82	15	6.6 ± 0.04	Cropland
Cropland	Halchter	1966	48	70	Sugar beet, wheat, barley	Haplic Luvisol	3	83	14	6.5 ± 0.1	Cropland
Cropland	Salzgitter	1966	47	90	Sugar beet, wheat	Haplic Luvisol	3	83	14	6.9 ± 0.03	Cropland
Cropland	Warberg	1966	48	65	Sugar beet, wheat, barley	Fragic Luvisol	3	80	17	6.0 ± 0.1	Cropland

Although SOC content and stocks in forest and cropland subsoil were found to be enhanced over the long-term through deep ploughing, total SOC stocks in deep-ploughed forest soil were not greater than in reference soil. This is contrary to the observations made for the deep-ploughed cropland soils^25^. An important mechanism contributing to SOC sequestration in deep-ploughed soil is continuous SOC accumulation in the newly formed topsoil. The recovery and build-up of new SOC-rich topsoil was much slower in forest than in cropland soil.

SOC stock differences between deep-ploughed and reference topsoils were twice as high in forest soil as in cropland soil indicating slower topsoil SOC accumulation in forests compared to croplands after deep ploughing (Supplementary Table 1). The average time since deep ploughing was longer at the cropland sites (on average 46 years, Table 1) than at the forest sites (on average 38 years, Table 1), resulting in a shorter SOC accumulation period for deep-ploughed forest topsoils. Nevertheless, even when dividing SOC stock differences by the number of years between deep ploughing and sampling, SOC accumulation rates in forest topsoils were smaller than in croplands. This may be due to the fact that in cropland soil, carbon inputs in the form of crop residues and leaf litter are directly incorporated into the mineral soil through regular tillage operations. In forest soil, on the contrary, aboveground litter first ends up in the forest floor and is thereafter only partly transferred into the mineral soil^31^. Because the forest sites studied here had acidic pH values (between 3 and 5) and *Pinus sylvestris* was one of the dominant tree species (Table 1), litter decomposition and SOC incorporation into the mineral soil has probably been slow and limited^31^. This supported by the observation that SOC stock differences in the L-horizon between deep-ploughed and reference soils were not significant while the F + H-horizon and topsoil had significantly less SOC in deep-ploughed than in reference soil (Fig. 2). This also indicated faster decomposition of litter in the deep-ploughed plots than in the reference plots, possibly related to less acid topsoil in the deep-ploughed plots because of admixture with previous subsoil material. However, the topsoil pH values were only slightly different in deep-ploughed and reference plots at the Schwenow forest site.

In the deep-ploughed forest topsoil, the amount of N needed to build up SOC stocks to a level comparable to that in reference topsoil would be between 0.09 and 0.74 Mg ha^−1^ (Supplementary Table 1). However, forests are usually not fertilised with mineral N, so the N input is mainly derived from atmospheric N deposition. For European forests, an average atmospheric N deposition of approximately 20 kg ha^−1^ yr^−1^ has been reported^32^. This shows that the maximum possible SOC sequestration in forest soil was restricted by the available N supply, whereas N restriction was less probable in cropland soil due to a 4- to 15-fold greater N input by mineral and organic fertilisers^25^. The build-up of SOC stocks in the near-surface forest topsoil, which were lowered because of mixing with SOC- and N-poor subsoil material as a result of deep ploughing, thus encounters a certain N limitation compared with cropland topsoil. We hypothesised that under such relatively N-poor conditions, there was enhanced mineralisation by microorganisms of organic matter that reached forest topsoil in order to obtain N (N mining^33^). Thus, N mining might have further slowed SOC accumulation.

Having discussed the effects on SOC stocks, the further paragraphs will now review the effect of topsoil burial through deep ploughing on SOC stability and its possible mechanisms. Topsoil burial by deep ploughing increased SOC stability at all study sites (Fig. 3). Higher stability to mineralisation has also been observed on SOC buried via depositional processes compared to reference surface soil SOC^34^. In the present study, we determined potential SOC stability via laboratory incubation under standardised temperature and soil moisture conditions. Thus, it can be concluded that the stability of buried SOC is not solely caused by environmental conditions at greater soil depth, such as temperature, oxygen or water limitations.

**Figure 7:**
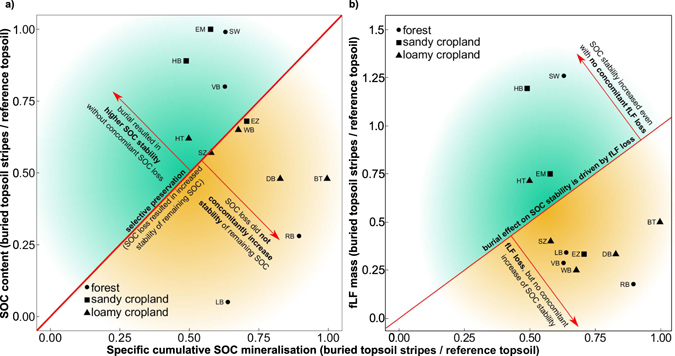
Correlation between ratio of specific cumulative SOC mineralisation in buried topsoil stripes to that in reference topsoil. (**a**) ratio of SOC content in buried topsoil stripes to that in reference topsoil as well as (**b**) ratio of fLF mass in buried topsoil stripes to that in reference topsoil. Sites abbreviations: LB - Lindenburg, RB -Rebberlah, SW - Schwenow, VB - Viborg, EZ - Elze, EM - Essem¨uhle, BT - Banteln, DB - Dr¨uber, HT - Halchter, SZ - Salzgitter and WB – Warberg.

Selective preservation of certain SOC fractions with higher stability^35^ could theoretically explain the preservation of SOC that has been buried for several decades. The labile fractions of SOC would then be mineralised leaving the most stable fraction as buried SOC. However, this mechanism could not fully explain the results obtained in the present study, because great SOC loss after burial did not concomitantly result in increased stability of the remaining SOC (Fig. 7a). Contrary to our expectations, at three sites (Essemühle, Hemmelsberg and Schwenow), we observed only slight losses of buried SOC. At the same time, stability of buried topsoil SOC was 50–60% higher than that in reference topsoil. This high SOC stability is most likely related to the land use history as heathland or peatland (Table 1). Former heathland soils have been observed to contain very stable SOC possibly related to a high content of hydrophobic and toxic substances for decomposers^25, 36, 37^. In contrast, at the Banteln (loamy cropland), Drüber (loamy cropland), Lindenburg (forest) and Rebberlah (forest) sites we observed an SOC loss of more than 50%, but only minor increases in SOC stability, with the maximum stability increase in the remaining SOC being 36%. These results underline that stability is not an intrinsic property of SOC, e.g. via poorly degradable compound classes, but might be controlled by environmental factors that are not yet fully understood for subsoil OC^10^. The large variability of the density fraction results could not be related to soil properties such as texture or pH, as these were not substantially variable within land use types (Table 1). The differences in texture between sandy and loamy cropland soils were also not consistently related to the difference in the fractionation results.

The fLF of SOC has previously been found to be the most easily mineralisable SOC fraction^38^, and it can be expected to be mineralised within one decade. However, our observations did not consistently show this trend, since at four out of 12 sites studied more than 50% of the fLF persisted in the buried topsoil stripes (Fig. 4). Surprisingly, we observed conflicting results with on the one hand, increased stability of buried SOC compared with the reference topsoil OC without a concomitant loss of fLF (Fig. 7b). This is similar to previous observations in buried colluvial soil of Canada, which were found to contain equal or greater mass of LF carbon than surface soil^34^. On the other hand, at eight out of 12 sites in the present study, fLF decreased upon burial by 50–80%, with only a slight increase in SOC stability of at most 42% (Fig. 7b). Thus, it can be concluded that the burial induced SOC stability was not driven by fLF loss with selective preservation of stabilised SOC. These findings underline that particulate organic matter (LF) at our sandy sites can be quite stable which is also confirmed by low ^14^C values (see below), likely due to the heathland history.

Higher fLF content in buried topsoil stripes than in reference topsoil was observed at the Schwenow forest site and the Hemmelsberg sandy cropland sites (Fig. 7 and Supplementary Fig. 1). At both sites more than 75% of SOC in buried topsoils was particulate organic carbon in the fLF fraction (Fig. 4). For Schwenow, the high fLF could be attributed to abundant roots in the buried topsoil stripes and related carbon inputs (Fig. 5b). At the same time, the Schwenow and Hemmelsberg sites showed a high degree of topsoil SOC preservation since burial (99% and 89%, respectively) and high SOC stability in the buried topsoil stripes (37% and 51% higher than in the reference topsoil). Under the assumption that roots are the main source of fLF at both sites, these findings indicate that (i) additional carbon inputs from roots growing in buried topsoil stripes do not lead to additional loss of the buried SOC via priming (stimulation of SOC mineralization after addition of fresh C input) and (ii) the roots themselves may promote total SOC storage in the subsoil. This is in line with findings that root-derived carbon persists over twice as long in soils before being decomposed than shoot-derived carbon^39^.

Roots grew preferentially in the buried topsoil stripes in the subsoil of deep-ploughed soil. We suggest that this is attributable to the higher organic matter content providing nutrients and water retention. This was confirmed by the visible presence of roots in deep-ploughed forest soil compared with reference soil (Fig. 5). Roots are a major source of subsoil SOC^39, 40^, both as exudates and in particulate form^41^. It has also been reported that subsoil loosening, another subsoil melioration option, promotes root proliferation into deeper soil layers^23^. In the present study, this could be confirmed for the forest sites but not for croplands because root biomass sampling at the cropland sites was conducted mainly during winter or after harvest. Also, cultivated crops were not particularly deep rooting plants (Table 1).

The depletion in Δ^14^C observed in buried topsoil stripes than in reference topsoil confirm the higher stability of buried SOC but also reflect the fact that input of carbon with dissolved organic carbon and roots into these soil fractions was drastically reduced due to burial. Buried SOC is relatively isolated compared to near surface SOC, which might be the key for its high stability. Buried topsoils were dominated by carbon that was older than the nuclear weapon testing in the 1960s and 1970s^42^ and recent carbon input from crop residues of the last years (negative Δ^14^C values). Reference topsoils at the forest sites and the loamy croplands mostly displayed positive Δ^14^C values (Fig. 7), indicating the influence of nuclear bomb test-derived carbon. In contrast, sandy croplands topsoils were dominated by old C, maybe remaining of the former land use as heathlands. The low ^14^C content at the Viborg forest site as compared to other forest sites is likely due to the former arable land use at this site just before deep ploughing.

In summary, deep ploughing can lead to increased SOC storage comprising two aspects: (i) greater stability of buried SOC and (ii) additional SOC accumulation in the “newly established” topsoil.

## Methods

### Study sites and sampling

Twelve experimental sites were selected for sampling, namely four forest sites and three cropland sites on sandy soils and five cropland sites on loamy soils (Table 1). Each site comprised a deep-ploughed plot and an adjacent reference, non-deep-ploughed plot with plot size 20 m by 40 m. All other site factors, such as forest and cropland management, soil characteristics, tree species and crops were equal or very similar in both plots (Table 1). Deep ploughing was conducted once 25 to 53 years before sampling, which represent the number of years in which a new topsoil was formed. Rebberlah was the only site that was not an experimental field site, but was partially deep-ploughed after a wildfire. The Lindenburg^43^ and Schwenow sites were clear-cut and partially deep-ploughed for experimental purposes regarding soil loosening and thus improvement of tree growth conditions. The Viborg site, located in Jutland, Denmark, is part of an experimental site studying different site preparation measures for afforestation of former arable land. The other three sites were located in northern and eastern Germany (Supplementary Table 1). The eight cropland sites were located in Northern Germany. At each site one field was partly deep ploughed. The remaining field was used as reference plot. All sites the topsoil was conventionally tilled using mouldboard ploughs.

We analysed four forest sites and eight cropland sites, five loamy and three sandy sites. Parts of the cropland sites data were also reported in a previous study^25^. All these twelve sites were sampled as described above. Soil sampling was conducted by taking five soil cores in each plot down to 100 cm to assess SOC stocks. These cores were divided into four depth increments: (1) topsoil (2) subsoil down to deep ploughing depth (Table 1), consisting of alternating buried topsoil stripes and subsoil stripes in the deep-ploughed plots (3) deep-ploughing depth + 10 cm and (4) deep subsoil down to 100 cm. Forest floor was sampled prior to coring with 25 cm by 25 cm metal frames separating horizons into L (undecomposed leaf litter) and F+H (partly decomposed organic matter). After drying at 65°, sieved roots and stones were weighed to obtain root biomass (Fig. 5) and fine soil mass. In addition, representative soil samples from each soil depth increment were taken for chemical analyses from soil profiles. This enabled separated sampling of the buried topsoil stripes.

### Chemical and microbiological analyses

Basic chemical and microbial characterisation was conducted for all twelve sites^25^. SOC mineralisation was assessed in a one year batch incubation at 22° of 100 g soil dry matter in triplicate gas-tight 250 mL glass flasks. Water content was initially adjusted to 60% of the water-holding capacity of each soil and gravimetrically readjusted periodically to the initial water content. CO_2_ production was measured at day 1, 3, 8, 14, 31, 127, 195, 269 and 365 after incubation start. CO_2_ concentration in sampled 20 mL vials was measured with a gas chromatograph (Series GC-2014; Shimadzu Deutschland GmbH, Duisburg, Germany). Specific CO_2_ production per g SOC as a cumulative sum of the total incubation year was computed.

Density fractionation^44, 45^ of SOC was performed by suspending 30 g soil dry matter in 120 mL of 1.6 g cm^−3^ sodium polytungstate (SPT) to separate a free light fraction (fLF). Dispersion by ultrasound with an energy input^46^ of 400 J ml^−1^ and resuspension in SPT was applied to obtain an occluded light fraction (oLF). A heavy fraction (HF) remained as sediment. Radiocarbon content in oLF and HF was assessed by acceleration mass spectrometry with preceding sample preparation^47^ and calibrated to Fraction Modern^48^.

### Calculations and statistics

Calculated SOC stocks^49^ (Mg ha^−1^) were corrected for different masses to enable comparison on an equivalent mass basis^50^. Due to the uneven and diagonal distribution of buried topsoil stripes, SOC contents were reported instead of SOC stocks when analysing buried topsoil stability in comparison with adjacent subsoil stripes and reference subsoil. Data analysis to identify significant differences between deep-ploughed and reference soils was performed with R^51^ version 3.3.1. Normality of data was first checked with the Shapiro-Wilk test. If data sets were normally distributed, differences were evaluated with paired Student’s t-tests. Otherwise, Wilcoxon Rank Sum and Signed Rank tests were applied. When repeated observations per site were made, i.e. sampling of five cores per plot in each site, linear mixed effect models using package nlme^52^ were computed with plot (reference and deep-ploughed) as fixed effect and site as random effect. If necessary, variances were weighed to ensure homoscedasticity. Correlations between stability indicators were examined with Spearman correlation tests. Fraction Modern values of ^14^C were converted to absolute Fraction Modern^42^ and then to Δ^14^C using R package SoilR^53^ version 1.1-23.

## Electronic supplementary material

Supplementary information
