# Supplementary material for: Stability of buried carbon in deep-ploughed forest and cropland soils - implications for carbon stocks
Source: Sci Rep. 2017 Jul 14;7:5511. doi: 10.1038/s41598-017-05501-y (PMC5511150; doi:10.1038/s41598-017-05501-y)

Supplementary information to the manuscript

Stability of buried carbon in deep-ploughed forest and cropland soils - implications for carbon stocks

by Viridiana Alcántara, Axel Don, Lars Vesterdal, Reinhard Well, Rolf Nieder

Supplementary Table 1: Topsoil C stocks difference in reference and deep ploughed soils (N=5 determined from soil coring). Absolute stock differences represent the subtraction of stocks in deep ploughed topsoils minus those in reference topsoils. Thus, positive values represent higher stocks in topsoil of the deep ploughed soils. Relative differences were calculated by dividing the stock in the topsoil of the deep ploughed by the stock in the reference topsoil. Values higher than 1 represent that the deep ploughed soils had higher stocks.

Land use	Site	Absolute C stock difference [Mg ha^{-1}]	Relative C stock difference	Absolute C stock difference relative to years since deep ploughing [Mg ha^{-1} yr^{-1}]
Forest	Lindenburg	-23.8±6.7	0.4	-0.6
Forest	Rebberlah	-3.4±2.7	0.9	-0.1
Forest	Schwenow	-8.5±3.0	0.7	-0.2
Forest	Viborg	-8.8±2.6	0.6	-0.4
Cropland	Elze	-8.5±2.9	0.8	-0.2
Cropland	Essemühle	-11.6±6.6	0.9	-0.3
Cropland	Hemmelsberg	-23.6±15.9	0.9	-0.7
Cropland	Banteln	0.1±2.7	1.0	0.0
Cropland	Drüber	-6.1±3.0	0.9	-0.1
Cropland	Halchter	0.3±1.2	1.0	0.0
Cropland	Salzgitter	0.5±2.5	1.0	0.0
Cropland	Warberg	-2.7±2.7	0.9	-0.1

Supplementary table 2: Site characteristics. MAT: mean annual temperature, MAP: mean annual precipitation

Site	Latitude, Longitude	MAT [°C]	MAP [mm]	Elevation [m above sea level]
Lindenburg	52°32’39“ N 9°34‘15“ E	9.2	700	64
Rebberlah	52°43‘31“ N 10°10.27“ E	8.8	730	88
Schwenow	52°9‘10“ N 14°2‘2“E	9.4	849	63
Viborg	56°24‘1“ N 9°19‘5“E	8.4	801	49
Elze	52°35'06"N 9°45'29"E	9.2	698	38
Essemühle	52°45'51"N 8°28'35"E	9.2	717	30
Hemmelsberg	53°04'60"N 8°19'40"E	9.2	750	10
Banteln	52°05'14"N 9°44'56"E	9.2	703	86
Drüber	51°45'28"N 9°54'22"E	8.9	687	154
Halchter	52°08'45"N 10°30'32"E	9.0	642	54
Salzgitter	52°04’12“N 10°27'18"E	9.1	647	116
Warberg	52°11'09"N 10°54'17"E	8.9	649	141

Supplementary figure 1: Relative mass proportion of bulk soil in SOC fractions (fLF: free light fraction, oLF: occluded light fraction, HF: heavy fraction)

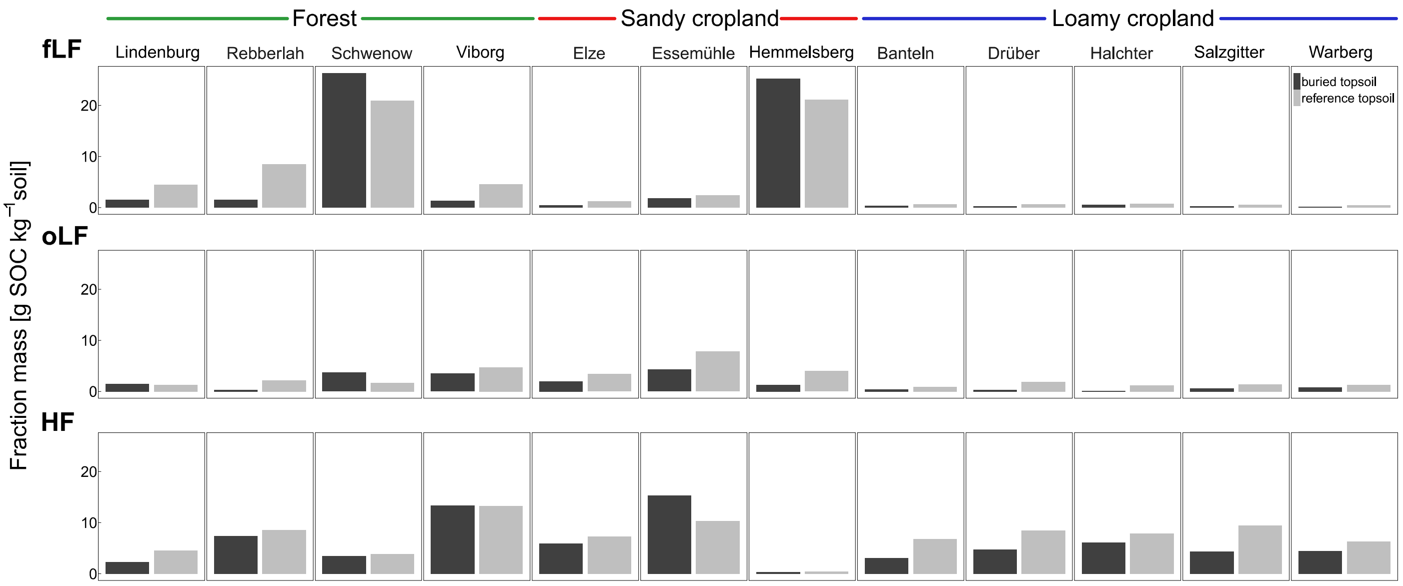

Supplement: Supplementary file 1 — Supplementary information [file 41598_2017_5501_MOESM1_ESM.pdf]
